# Association of micronutrient status with thyroid function in adolescent Afghan refugees; a cross-sectional study

**DOI:** 10.1186/s13044-025-00239-6

**Published:** 2025-06-03

**Authors:** Saima Shaheen, Muhammad Shahzad, Nabila Sher, Muhammad Shabbir Khan, Khalid Iqbal, Habab Ali Ahmad, Simon C Andrews

**Affiliations:** 1 Department of Biochemistry, Khyber Girls Medical College, Peshawar, Pakistan; 2 Faculty of Dentistry, Zarqa University, Zarqa, Jordan; 3 Institute of Basic Medical Sciences, Khyber Medical University, Peshawar, Pakistan; 4 Department of Biochemistry, Khyber Medical College, Peshawar, Pakistan; 5 Department of Biomedical Sciences, Pak-Austria Fachhochschule Institute of Applied Science and Technology (PAF-IAST), Haripur, Pakistan; 6 School of Biological Sciences, Health and Life Sciences Building, University of Reading, Reading, RG6 6EX UK

## Abstract

Micronutrients play a crucial role in several metabolic processes including thyroid hormone metabolism and functions. The current study aimed to assess the associations between thyroid hormone levels and micronutrient status in a cohort of adolescents Afghan refugees residing in a refugee camp in Pakistan. A randomised, community based, cross-sectional study design was employed to recruit 206 adolescent (both male and female) Afghan refugees aged 10–19 years. Sociodemographic data, anthropometric assessments and blood samples were collected using standard methods. Serum vitamins, minerals and thyroid hormones levels were assessed using ELISA, electrochemiluminescence and inductively coupled plasma mass spectrometry (ICP-MS) respectively. Overall results showed the median levels of T3 and TSH were significantly higher (*p* < 0.05) in younger adolescents (10–14 years) compared to 15–18 year olds while T4 was significantly higher in boys compared to girls. Correlational analysis between serum micronutrients status (vitamin D, vitamin B12, ferritin, folate, zinc, copper, selenium) and thyroid hormones revealed significant relationships in different age groups. Overall, vitamin D exhibited a statistically significant positive correlation with T4 (*r* = 0.279) in the combined, younger (*r* = 0.277) and older (*r* = 0.319) age groups. In contrast, a statistically significant but negative correlation was observed when zinc levels were compared with T3 (*r*=-0.288) in the older age group and with T4 (*r*=-0.195) in the younger age group. In conclusion, micronutrients status, especially vitamin D and zinc, have important implications for thyroid health and thereby require close monitoring in any thyroid deficiency related disorders in vulnerable population such as refugees.

Clinical trial number: Not applicable.

**Supplementary Information:**

The online version contains supplementary material available at 10.1186/s13044-025-00239-6.

## Introduction

Micronutrient deficiencies are generally caused by inadequate intake of essential micronutrients (vitamins and minerals) and represent a major global public health burden. This problem is largely limited to developing nations and socioeconomically disadvantaged populations. Estimated reports from the World Health Organization (WHO) indicate that over 2 billion people worldwide suffer the physiological consequences of micronutrient insufficiency [1]. Micronutrient insufficiencies are widespread causes of suppressed immune function, disturbed metabolism, impaired physical and cognitive development in children, and increased risk of chronic conditions such as cancer and cardiovascular diseases [2]. Optimal metabolism in humans is predicated on adequate supplies of micronutrients that act as enzyme cofactors and structural components for macromolecules. One of the major human-health impacts of micronutrient availability is thyroid metabolism and function [3].

In humans, the thyroid gland plays an important role in growth and development through maintenance of homeostasis and by supporting the normal function of the cardiovascular, reproductive and nervous systems [4]. Thyroid function is primarily regulated by the hypothalamic-pituitary-thyroid axis through the action of thyroid-stimulating hormone (TSH), triiodothyronine (T3) and thyroxine (T4). Thyroid hormones are crucial for cellular development, differentiation, growth, and regulation of protein, lipid and carbohydrate metabolism in nearly all tissues [5]. Thyroid hormone signaling is also essential for normal growth and maturation of organs such as the brain, lung, heart, skeletal muscle and bone. However, the production of inappropriate amounts of thyroid hormones results in hypothyroidism or hyperthyroidism which have devastating consequences on human health. Hyperthyroidism causes weight loss, heat intolerance and rapid heartbeat (tachycardia) while hypothyroidism is characterized by weight gain, feeling cold (cold intolerance), constipation, enlargement of the thyroid gland (goiter) and slowed metabolism (metabolic disruptions) [6]. Although the exact etiology and pathogenesis of thyroid disorders are not known, a role for micronutrients and trace elements is frequently suggested. Several micronutrients, especially trace elements, are found in higher concentration in the thyroid gland than in any other tissue in the body [7] and are essential for thyroid hormone synthesis, metabolism and function. Micronutrient status is also a key determinant of risk and severity of autoimmune thyroid disorders (AITD) as both micronutrient deficiency and excess can promote autoimmune attack on the thyroid gland. Indeed, epidemiological studies indicate an increased susceptibility to pathogenic thyroid dysfunction is linked to the dietary availability of the micronutrients iodine, iron, selenium, copper, zinc, and vitamins B12 and D [8].

Micronutrients, especially trace elements, are crucial to maintain thyroid metabolism and function. Mounting research evidence suggests that iron and iodine are closely related to thyroid metabolism and, as a result, iron deficiency is frequently associated with thyroid dysfunctions in humans [9]. Se deficiency has been repeatedly associated with increased risk of AITD, such as Graves’ disease and Hashimotos’s thyroiditis, and is also linked with exacerbated developmental hypothyroidism primarily caused by iodine deficiency [10]. In-vivo studies in animal models demonstrate that inadequate zinc levels impair thyroid hormone production, potentially causing hypothyroidism, developmental abnormalities and compromised immunity [11]. Thyroid hormones are also reported to modulate Zn homeostasis by regulating its intestinal absorption and renal reabsorption in rats [12]. In humans, the finding of a strong correlation between tissue levels of thyroid hormones and zinc suggests an intimate bidirectional relationship between the two [13]. Furthermore, zinc supplementation in humans results in a significant increase in plasma TSH, T3 and T4 levels [14].

Although the impact of micronutrients on thyroid functions has been frequently reported in the literature, such studies have been mostly focused on thyroid disease patients, children and pregnant women [15, 16]. Until now, little or no research investigating the relationship between micronutrient status and thyroid function has been conducted in populations that are at high risk of malnutrition, such as refugees. Refugee populations living in refugee camps, are particularly vulnerable to malnutrition due to factors like chronic food insecurity, limited access to diverse diets, and prolonged exposure to stress and adverse living conditions [17]. Consequently, malnutrition (especially deficiencies of essential micronutrients like iodine, selenium, and zinc) render these population at higher risk of endocrine and metabolic disorders, including thyroid dysfunction [18]. Therefore, this cross-sectional study aimed to investigate the associations between thyroid hormone levels and micronutrient status in a cohort of adolescent Afghan refugees residing in a refugee camp in Pakistan.

## Methods

### Study design and population

This cross-sectional study analysed 206 adolescent (both male and female) Afghan refugees (aged 10–19 years) residing in Khazana refugee camp, the largest refugee camp (5000 residents) in Peshawar, Pakistan. All participants shared similar regional backgrounds, dietary limitations and access to healthcare, making them an appropriate cohort for examining micronutrient levels in relation to thyroid function in a nutritionally vulnerable population. They were recruited by a non-probability, consecutive sampling method in a study assessing the nutritional status of apparently healthy adolescents [19]. Pregnant or lactating women, those physically or mentally handicapped and those who were on medication (antibiotics, nutritional supplements, pre and probiotics) at the time of data collection or in the past two months were excluded from the study. Participants with incomplete responses in the questionnaire data (*n* = 2), or missing plasma micronutrients data (*n* = 17) or thyroid hormone levels (*n* = 16) were also excluded from analysis yielding a final sample of 182 participants (91 boys and 91 girls). An information sheet containing the detailed procedures and purpose of the study in an easy to understand, local language (Pashto and Dari) was provided to all the participants before signing a written informed consent. Ethical and administrative approval for the study was provided by Khyber Medical University and the Peshawar Afghan Commissionerate (Peshawar), respectively.

### Data and samples collection

Participant demographic and socioeconomic status information was collected by an interviewer-administered, structured questionnaire. Trained data collectors measured the height and weight of the participants using standard procedures. The body mass index (kg/m^2^) was calculated using the participants’ height and weight. Blood samples were drawn between 08:00 and 10:00 a.m. to account for diurnal variations in micronutrient and hormone levels. Plasma and serum were separated, transported to the main lab within 4 h and stored at -80 °C prior to analysis.

### Laboratory assays

Complete blood counts were performed on freshly collected whole blood samples using a Sysmex automatic haematology analyzer (XP-100, Jalan Tukang, Singapore). Vitamin D status was assessed using a 25-OH vitamin D Diasorin radioimmunoassay ELISA kit (Euroimmun, Germany) while ferritin, vitamin B12 and folate were assessed using the Abbott Architect i2000 analyser (Abbott Diagnostics, Zug, Switzerland). Thyroid function tests were performed to measure the serum levels of total T3, T4 and thyroid stimulating hormone (TSH) using an Elecsys electrochemiluminescence based assay on a Cobas e 411 analyser (Roche Diagnostics, Germany). The levels of iron, zinc, selenium and copper were determined by reconstituting freeze-dried plasma samples in Milli-Q (18 MΩ) water and subjecting it to inductively coupled plasma mass spectrometry analysis (ICP-MS; Thermo Fisher Scientific iCAPQ, Bremen, Germany).

### Statistical analysis

Age is reported as mean (SD) and age categories, educational status, family size and female household head educational status are reported as frequencies (percentage). This study used tertiles to categorize serum levels of T3, T4, and TSH to facilitate comparison across low, middle, and high levels of thyroid hormones. This tertile-based classification allows for assessing variations in micronutrient concentrations across different thyroid hormone ranges without assuming normality in data distribution, making it suitable for evaluating nonlinear relationships in micronutrient-thyroid interactions. Micronutrient concentrations are described as medians (Q1 and Q3) by tertiles for T3, T4 and TSH. Quantile regression was used to investigate association among thyroid hormones (T3, T4 and TSH).

Statistical analyses considered age, gender and BMI as demographic variables. Gender distribution was assessed across the tertile groups, which did not reveal statistically significant differences but indicated a trend. For clarity, analyses were adjusted to control for gender effects on micronutrient-thyroid associations, minimizing any potential confounding influence from gender differences within tertiles. Due to non-normal distribution of the micronutrient levels, the linear trends of these biomarkers across ordered categories of thyroid hormones (T3, T4 and TSH) were assessed using the Jonckheere Terpstra Test. Correlation between biomarkers and thyroid hormones were assessed using Spearman rank correlation. All data analysis was conducted using SAS Version 9.4.

## Results

### General characteristics of the study participants

General characteristics of the 206 study participants, 103 males and 103 females are presented in Table 1. Participants were divided into two age groups, 10–14 (63.7%) and 15–19 (36.3%) years. Background characteristics of the study participants were described previously and are thus provided here in supplementary Table S1.

**Table 1:** Association of thyroid hormone levels with age, sex and BMI

Characteristics	T3	T4	TSH
All ages			
Age (Categories)	0.22 (0.05, 0.43) *	0.2 (-0.75, 0.81)	0.46 (0.01, 0.74) *
Sex (Gender = 1)	0.12 (-0.01, 0.26)	1.29 (0.64, 1.94) *	0.07 (-0.27, 0.34)
BMI (kg/m^2^)	0.001 (-0.04, 0.03)	0.1 (-0.08, 0.2)	0.04 (-0.03, 0.11)
**Age Group (10–14 Years)**			
Sex (Gender = 1)	0.18 (0, 0.29) **	1.32 (0.36, 2.04) *	-0.11 (-0.47, 0.42)
BMI (kg/m^2^)	0.02 (-0.04, 0.04)	0.17 (-0.06, 0.37)	0.08 (-0.04, 0.13)
**Age Group (14–19 Years)**			
Sex (Gender = 1)	0.16 (-0.33, 0.29)	1.26 (0.15, 2.21) *	0.29 (-0.05, 0.59)
BMI (kg/m^2^)	-0.01 (-0.07, 0.02)	0.001 (-0.18, 0.19)	-0.01 (-0.09, 0.05)

*Coefficient estimates with 95% CI from quantile regression modeling median of T3*,* T4 and TSH to investigate association with age*, sex and BMI. * p-value < 0.05, **p-value = 0.05

### Association between demographic and nutritional status, and thyroid function biomarkers

We first assessed whether there is any relationship between demographic characteristics (age, gender), nutritional status (BMI) and median levels of thyroid hormones among the participants (Table 1). Overall, the results show that median levels of T3 and TSH are significantly higher in 10–14 years as compared to 15–18 years. Although no significant association was observed for median levels of T4 with age, T4 concentration was significantly higher in boys compared to girls. Stratified analysis by age group showed that T4 levels are significantly higher among boys in both age groups while T3 was high only in the younger adolescent group (10–14 years). However, no significant association was observed between thyroid hormones level and BMI (Table 1).

### Association between plasma micronutrients levels and thyroid function biomarkers

To assess the association of selected micronutrients with thyroid function biomarkers, the median concentration of the micronutrients was divided into three equivalently sized tertiles based on serum T3, T4 and TSH levels. No statistically significant trend was observed between serum micronutrient levels across the three tertiles based on serum T3 levels (Table 2). However, stratified analysis by age showed a significantly decreasing trend in serum ferritin and zinc levels from across tertile 1.

**Table 2:** Comparison of micronutrientstatus and T3 levels in adolescent Afghan refugees. Comparison was achieved by dividing participants into three equally-sized tertiles according to T3 levels

Characteristics		T3			
	***Overall***	***Tertile 1***	***Tertile 2***	***Tertile 3***	***P-trend***
Median (Q1, Q3) of T3		1.05 (0.93, 1.16)	1.42 (1.33, 1.51)	1.79 (1.67, 2.06)	
All age groups					
Ferritin (ng/mL)	39 (27, 61.4)	48.0 (29.5, 79.6)	35.0 (24.6, 49.0)	40.6 (26.54, 59.45)	0.09
Folate (ng/mL)	4.4 (3.4, 6.1)	4.6 (3.1, 6.6)	4.3 (3.6, 5.8)	4.2 (3.6, 6)	0.82
Vitamin B12 (pg/mL)	219 (157, 348)	228 (167, 392)	220.0 (155.0, 349.0)	205.0 (161.0, 300.0)	0.29
Vitamin D (ng/mL)	22.3 (16.2, 27.5)	20.4 (13.9, 25.1)	25.3 (19.3, 28.9)	21.5 (15.7, 26.9)	0.17
Zinc (µg/L)	787 (619, 932)	809 (637, 939)	784.5 (623.8, 934.4)	758.6 (593.7, 891.7)	0.33
Copper (µg/L)	951 (791, 1147)	936 (771, 1217)	970.2 (827.1, 1134.6)	951.4 (797.1, 1082.3)	0.56
Selenium (µg/L)	71.1 (50.5, 91.3)	72.4 (55.4, 93.3)	75.2 (50.5, 96.1)	62.9 (45.1, 84.2)	0.10
Age (10–14)					
Ferritin (ng/mL)	37.7 (27.8, 51.6)	39.4 (29.5, 60.3)	35.5 (26.9, 44.7)	40.7 (27.2, 59.5)	0.95
Folate (ng/mL)	4.7 (3.6, 6.1)	5.3 (4, 6.8)	4.5 (3.625, 5.7)	4 (3.6, 5.7)	0.08
Vitamin B12 (pg/mL)	209 (155, 310)	209.5 (157, 337)	213.5 (155.5, 302.5)	205.0 (154.0, 287.0)	0.56
Vitamin D (ng/mL)	24.1 (7.3)	22.9 (6.9)	24.1 (7.5)	25.0 (7.5)	0.33
Zinc (µg/L)	789.6 (605.6, 934.4)	773.2 (576.6, 900.7)	798.4 (631.2, 933.7)	789.6 (642.6, 949.4)	0.46
Copper (µg/L)	992 (824.6, 1146.8)	933.3 (765.7, 1188.6)	1014.1 (837.4, 1146.4)	987.7 (824.6, 1118.3)	0.78
Selenium (µg/L)	72 (48.4, 91.4)	73.4 (49.3, 102.5)	70.5 (46.3, 95.5)	68.5 (54.2, 86.2)	0.48
Age (15–18)					
Ferritin (ng/mL)	48.3 (23.5, 79.8)	66.1 (31.3, 105.4)	34.1 (17.5, 57.3)	35.2 (23.1, 56.5)	**0.04**
Folate (ng/mL)	4 (3.1, 6.3)	4 (2.85, 6.05)	4 (3, 6.2)	4.8 (3.6, 6.7)	0.20
Vitamin B12 (pg/mL)	244 (170, 400)	250.0 (174.5, 428.5)	250.0 (148.0, 443.0)	207.0 (175.0, 349.5)	0.59
Vitamin D (ng/mL)	17.3 (12, 25.1)	16.8 (12, 23.65)	24.4 (14.9, 30.4)	14.7 (11.35, 17.35)	0.91
Zinc (µg/L)	784.5 (621.4, 905.2)	838.3 (713.5, 969.1)	697.2 (623.8, 1011.0)	632.9 (539.5, 746.0)	**0.004**
Copper (µg/L)	926.1(731.2, 1107.3)	947.3 (773.6, 1235.0)	941.1 (761.1, 975.4)	817.1 (615.5, 991.4)	0.09
Selenium (µg/L)	70.6 (51.5, 89.5)	71.1 (55.6, 88.4)	85.9 (53.0, 100.2)	42.9 (37.7, 71.5)	0.08

*Mean and range of T3 levels in each tertile were: Tertile 1 (mean = 2.0 µg/dL; range = 1.5–2.5 µg/dL) Tertile 2 (mean = 3.0 µg/dL; range = 2.6–3.5 µg/dL) and Tertile 3 (mean = 4.0 µg/dL*,* range* of *3.6–4.5 µg/dL). Significant differences are indicated in bold. A P-trend value of < 0.05 indicates that the the observed trend is significant (increase or decrease of the micronutrient indicator across the T3 tertials) and was determined using the Jonckheere-Terpstra test*

The association of serum micronutrient levels with serum T4 levels is shown in Table 3. Vitamin D levels exhibit an increasing trend across T4 level tertiles 1–3. Vitamin D concentrations were higher in tertile 3 than tertiles 1 and 2, both in the combined age group and the older age group, but for the lower age group the tertile 3 levels were only higher than those of tertile 1. Vitamin D levels were also 1.3-fold higher in younger than older adolescents. An opposite trend was seen for zinc where levels were significantly (1.1-fold) higher for the younger age group in tertile 1 than in tertiles 2 and 3, and thus zinc levels display a clear negative correlation with T4 levels. In older adolescents (15–18 age group), selenium levels were significantly lower in tertile 1 compared to tertiles 2 and 3, and a positive correlation between selenium and T4 was apparent in this cohort.

**Table 3:** Comparison of micronutrient status and serum T4 levels in adolescent Afghan refugees. Comparison was achieved by dividing participants into three equally-sized tertiles according to T4 levels

Characteristics		T4			
	***Overall***	T1	T2	T3	***P-value***
Median (Q1, Q3)		4.7(3.74,5.7)	6.89(6.55,7.4)	9.05(8.52,10.45)	
All age groups					
Ferritin (ng/mL)	39 (27, 61.4)	39.1 (25.6, 62.6)	37.7 (27.0, 59.5)	39.4 (27.8, 57.3)	0.92
Folate (ng/mL)	4.4 (3.4, 6.1)	4.6 (3.6, 6.1)	4.6 (3.4, 6.3)	4.3 (3.3, 5.7)	0.41
Vitamin B12 (pg/mL)	219 (157, 348)	208.5 (159, 313)	244.0 (150.0, 350.0)	224.0 (158.0, 342.0)	0.65
Vitamin D (ng/mL)	22.3 (16.2, 27.5)	20.5 (15.0, 25.2)	21.1 (13.9, 27.7)	25.4 (20.0, 30.0)	**0.003**
Zinc (µg/L)	787.1 (618.6, 932.1)	805.5 (602.6, 1077.3)	806.1 (639.5, 933.1)	758.6 (593.7, 877.8)	0.16
Copper (µg/L)	951.5 (790.8, 1146.7)	928.5 (719.6, 1158.4)	970.2 (828.1, 1153.3)	956.3 (837.9, 1107.1)	0.65
Selenium (µg/L)	71.1 (50.5, 91.3)	69.4 (48.3, 95.7)	69.9 (51.5, 84.4)	73.7 (52.3, 95.0)	0.53
Age (10–14)	13.1 (12.4, 13.8)	13.1 (12.25, 13.85)	13.1 (12.3, 13.7)	13.1 (12.5, 13.7)	
Ferritin (ng/mL)	37.7 (27.8, 51.6)	35.8 (24.1, 51.4)	37.4 (29.4, 52.1)	39.4 (27.8, 54.7)	0.64
Folate (ng/mL)	4.7 (3.6, 6.1)	4.8 (3.6, 6.35)	4.8 (3.6, 5.95)	4.3 (3.6, 5.7)	0.66
Vitamin B12 (pg/mL)	209 (155, 310)	207.0 (155.5, 260.0)	259.0 (148.0, 316.0)	205.0 (156.0, 310.0)	0.68
Vitamin D (ng/mL)	24.1 (7.3)	22.0 (6.2)	24.5 (7.8)	25.8 (7.6)	**0.02**
Zinc (µg/L)	789.6 (605.6, 934.4)	884.0 (661.8, 1274.9)	798.4 (630.1, 941.3)	748.1 (593.7, 872.0)	**0.03**
Copper (µg/L)	992 (824.6, 1146.8)	971.6 (763.0, 1173.6)	999.5 (839.2, 1132.3)	974.6 (837.9, 1130.9)	0.96
Selenium (µg/L)	72 (48.4, 91.4)	78.9 (48.0, 109.7)	61.9 (45.4, 79.9)	72.5 (59.9, 87.5)	0.49
Age (15–18)					
Ferritin (ng/mL)	48.3 (23.5, 79.8)	60.7 (27.7, 92.2)	48.3 (18.06, 63.7)	44.3 (26.1, 82.6)	0.48
Folate (ng/mL)	4 (3.1, 6.3)	4.4 (3.3, 5.6)	4 (3.3, 6.6)	3.3 (2.6, 6.2)	0.29
Vitamin B12 (pg/mL)	244 (170, 400)	223.5 (178.5, 393.5)	227.0 (170.0, 400.0)	304.0 (170.0, 443.0)	0.75
Vitamin D (ng/mL)	17.3 (12, 25.1)	15.3 (12, 22.2)	13.5 (11.5, 24.5)	22.9 (17.3, 27.5)	**0.05**
Zinc (µg/L)	784.5 (621.4, 905.2)	724.7 (499.0, 838.3)	821.9 (657.1, 909.1)	783.8 (551.9, 941.0)	0.36
Copper (µg/L)	926.1 (731.2, 1107.3)	781.0 (630.7, 1081.0)	937.7 (814.6, 1217.0)	944.7 (731.2, 975.4)	0.46
Selenium (µg/L)	70.6 (51.5, 89.5)	58.2 (50.2, 71.1)	75.3 (60.6, 93.3)	84.9 (41.6, 111.1)	**0.02**

Vitamin D levels were also significantly associated with TSH levels in the younger age group (Table 4). Lower vitamin D levels were correlated with lower TSH levels, which is opposite to the trend observed between vitamin D and T4.

**Table 4:** Comparison of micronutrient status and TSH levels in Afghan adolescent refugees. Comparison was achieved by dividing participants into three equally-sized tertiles according to TSH levels

Characteristics		TSH			
	***Overall***	T1	T2	T3	***P-value***
Median		1.18 (0.96,1.33)	1.7 (1.54, 1.87)	2.5 (2.28, 2.93)	
All age groups					
Ferritin (ng/mL)	39 (27, 61.4)	40.0 (28.0, 62.1)	44.0 (33.8, 67.8)	31.7 (20.7, 52.7)	0.14
Folate (ng/mL)	4.4 (3.4, 6.1)	4.6 (3.4, 6.65)	5 (3.6, 6.1)	4 (3.5, 5.2)	0.12
Vitamin B12 (pg/mL)	219 (157, 348)	220.5 (174, 352.5)	230.0 (165.0, 355.0)	209.0 (145.0, 305.0)	0.27
Vitamin D (ng/mL)	22.3 (16.2, 27.5)	22.2 (16.35, 28.6)	23.0 (14.8, 27.6)	21.5 (17.1, 26.2)	0.39
Zinc (µg/L)	787.1 (618.6, 932.1)	780.1 (631.2, 899.6)	748.1 (593.7, 877.8)	811.8 (644.4, 956.4)	0.42
Copper (µg/L)	951.5 (790.8, 1146.7)	1019.4 (814.0, 1221.3)	934.7 (761.1, 1077.9)	960.3 (828.1, 1132.9)	0.45
Selenium (µg/L)	71.1 (50.5, 91.3)	74.0 (52.5, 96.1)	67.5 (48.4, 86.2)	71.0 (49.9, 86.4)	0.47
Age (10–14)					
Ferritin (ng/mL)	37.7 (27.8, 51.6)	40.7 (28.8, 62.0)	40.7 (32.3, 51.2)	32.5 (21.7, 49.9)	0.08
Folate (ng/mL)	4.7 (3.6, 6.1)	4.8 (3.6, 6.6)	5.1 (4, 6.1)	4.1 (3.6, 5.2)	0.08
Vitamin B12 (pg/mL)	209 (155, 310)	209.0 (176.0, 292.0)	220.0 (157.0, 315.0)	207.0 (145.0, 305.0)	0.68
Vitamin D (ng/mL)	24.1 (19.3, 28.3)	26.8 (21.1,31.3)	24.7 (17.1,28.9)	22.4 (18.4, 26.9)	**0.03**
Zinc (µg/L)	789.6 (605.6, 934.4)	798.3 (642.6, 933.1)	724.9 (593.7, 884.0)	803.4 (600.1, 956.4)	0.77
Copper (µg/L)	992 (824.6, 1146.8)	1102.0 (821.5, 1200.8)	951.6 (837.9, 1118.3)	963.5 (824.6, 1132.9)	0.21
Selenium (µg/L)	72 (48.4, 91.4)	78.5 (64.0, 97.6)	63.2 (46.8, 86.9)	68.2 (47.9, 86.3)	0.07
Age (15–18)					
Ferritin (ng/mL)	48.3 (23.5, 79.8)	37.5 (18.1, 62.2)	63.6 (35.3, 88.1)	23.0 (11.6, 130.0)	0.55
Folate (ng/mL)	4 (3.1, 6.3)	4.5 (3.1, 6.8)	4.1 (2.6, 6.2)	3.8 (3.1, 4.6)	0.30
Vitamin B12 (pg/mL)	244 (170, 400)	254.0 (172.0, 395.0)	264.5 (179, 482)	210.0 (147.0, 350.0)	0.64
Vitamin D (ng/mL)	17.3 (12, 25.1)	16.3 (12, 23.2)	20.5 (12.6, 25.7)	13.4 (11.2, 19.2)	0.65
Zinc (µg/L)	784.5 (621.4, 905.2)	754.2 (618.6, 885.4)	793.6 (551.9, 876.0)	878.9 (697.2, 969.1)	0.33
Copper (µg/L)	926.1 (731.2, 1107.3)	937.7 (751.5, 1241.1)	804.3 (642.4, 975.4)	955.2 (851.8, 1156.0)	0.60
Selenium (µg/L)	70.6 (51.5, 89.5)	61.4 (45.1, 89.0)	71.7 (52.1, 85.9)	80.3 (69.9, 95.8)	0.15

**Fig. 1:**
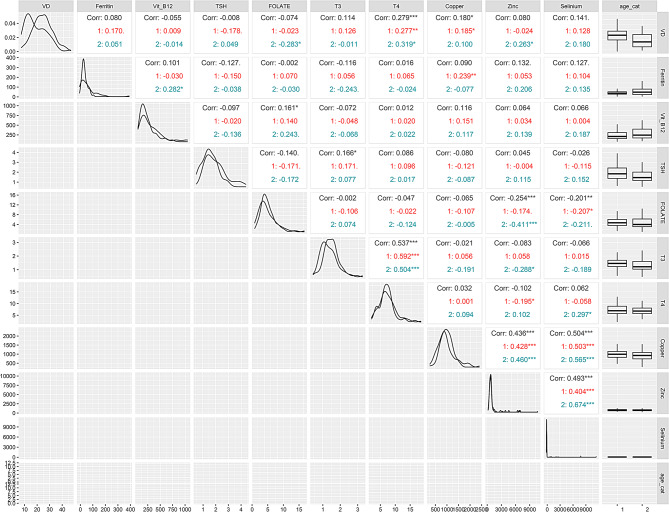
Corretion coefficient, data distribution and scatter plot of the micronutrients with T3, T4 and TSH by gender (all genders). The upper right triangle present correlation coefficients and their statistical significance values are represented by asterisk. Black color represents significance of overall correlation while red and navy-blue color represent correlation with in the younger (age 10–14 years) and older (15–19 years) adolescents group respectively. * indicate *P* ≤ 0.05, ** indicate *p* ≤ 0.01 and *** indicate *P* ≤ 0.001.The diagonals indicate distribution of each parameter and lower triangular matrix indicate scatter plot. The right side column represent the box plot distribution of each parameter based on age groups while the lower horizontal row indicate histogram

Analysis of the relationship between serum micronutrients and thyroid hormone levels, on a continuous scale (Fig. 1), indicates that vitamin D exhibits a statistically significant positive correlation with T4 (*r* = 0.279) in the combined, younger (*r* = 0.277) and older (*r* = 0.319) age groups. In contrast, a statistically significant but negative correlation was observed when zinc levels were compared with T3 (*r*=-0.288) in the older age group and with T4 (*r*=-0.195) in the younger age group.

## Discussion

To our knowledge, this is the first study evaluating the association between thyroid hormone levels and micronutrient status in Afghan adolescent refugees. Thyroid dysfunction is a worldwide public health problem, the etiology of which is mostly unknown. Clinically, thyroid dysfunctions mainly involve subclinical thyroid function disorder and is identified by assessment of the levels of T3, T4 and TSH in serum along with other routine clinical investigations. In humans, thyroid metabolism depends on processes which are influenced by micronutrients status [8]. Although, iodine is the key micronutrient in thyroid hormone biosynthesis [20], other micronutrients are also involved in maintaining normal thyroid function [16]. For example, selenium (Se) is a constituent of three iodothyronine deiodinase enzymes that catalyze the conversion of prohormone thyroxine (T4) to the active hormone 3,3′,5-triiodothyronine (T3). Thus, Se plays a direct role in the metabolism and activation of thyroid hormones [21]. Zinc is another essential micronutrient with a well-established role in thyroid hormone biosynthesis, metabolism and signaling. Zn is an essential structural component and catalytic cofactor for numerous enzymes involved in thyroid hormone biosynthesis [22], playing a role in thyrotropin-releasing hormone (TRH) production. TRH stimulates release of thyroid stimulating hormone (TSH) from the pituitary, which in turn acts on the thyroid to promote thyroid hormone production [23]. Thus, micronutrient deficiency can exacerbate thyroid disorders [24], which is concerning given that almost one third of the global population suffer from deficiency in one or more micronutrients.

Our study indicates that there are age and gender dependent differences in thyroid hormone levels among adolescent Afghan refugees. Specifically, T3 and TSH were significantly higher in younger (10–14 years) than older adolescents (15–18 years) while T4 levels were significantly higher in boys compared to girls. These observed age-related changes in thyroid hormone levels align with findings from previous studies, such as those conducted on Danish (aged 6–18 years) and Indian (aged 6–17 years) cohorts of school age children, where the median values of free T3 and T4 decreased with increasing age [25, 26]. However, our cohort is distinct in being composed solely of refugees, a population that may experience unique environmental and nutritional challenges exacerbating these age-related changes. The age-related changes were more obvious in young children with convergence to adult like ranges after age 15 years and above. Similarly, higher levels of thyroid hormones in boys than girls, as found in our study, has also been reported previously [27]. It is important to note that these differences could be influenced by factors not fully explored in this study, such as variations in physical activity, diet and exposure to environmental stressors like chronic malnutrition or psychosocial stress, which are prevalent in refugee populations. However, it should be noted that the age and gender-based changes in thyroid hormones levels may also be influenced by other factors, such as ethnicity, body mass index and methodological approaches to measure thyroid parameters [28]. These variables are important for understanding the full spectrum of influences on thyroid function and should be considered when interpreting findings especially in a diverse and vulnerable population such as refugees.

The current study reports an association between selected micronutrients and thyroid function, as reported previously. For example, a positive association was observed between vitamin D and both TSH and T4 levels in younger adolescents aged 10–14 years. These findings are in concordance with previous studies reporting significantly low levels of TSH and T4 in vitamin D deficient individuals aged 12 to 18 years [29]. Vitamin D deficiency has been implicated in autoimmune thyroid disorders including Hashimoto’s thyroiditis and Graves`s disease [30]. Although, the exact mechanism by which vitamin D deficiency impairs thyroid functions is unknown, experimental evidence on a mouse model suggests that vitamin D exerts its effect by increasing the mRNA expression levels of the de-iodinase 2 gene, which encodes an enzyme necessary for conversion of T4 to T3 [31]. Other possible mechanisms include suppression of TSH-stimulated adenylyl cyclase activity and enhanced iodine uptake [32]. Several clinical trials have investigated the impact of vitamin D supplementation on thyroid function [33]. Much research has reported a significant reduction in anti-thyroid antibodies following vitamin D supplementation with little or no effect on thyroid hormones levels [30]. However, a larger clinical trial encompassing 11,017 participants (mean age 48 years, ranging from 18-95 years, and 58% female), receiving vitamin D supplementation for 12 months, reported decreases in serum TSH, anti-TPO, anti-TG and TG levels over time, and increases in serum FT3 and FT4, leading to reduction in hypothyroidism and thyroid autoimmune disorder [34].

Among the trace elements assessed, zinc exhibited a significant negative correlation with serum T3 and T4, and a positive, but non-significant, correlation with serum TSH levels. While this observation aligns with the previous findings [35], it is important to note that other studies (e.g., [36]) reported either no correlation or a positive correlation between zinc levels and thyroid hormones. These differences in findings highlight the need for further investigation into the specific mechanisms through which zinc interacts with thyroid hormones. Unlike the consistent effect observed for other trace elements, zinc’s influence on thyroid function appears to be complex and potentially context dependent. Zinc contributes to thyroid metabolism through its role in the synthesis of the thyrotropin-releasing hormone (TRH) in the hypothalamus, as cofactor for deiodinase I and II enzymes that help in conversion of T4 to T3, or as structural component of the T3 receptor [14, 37].

Additionally, our study showed a positive association between T4 status and selenium levels. Selenium is a critical component of the deiodinase enzymes, which are responsible for the conversion of thyroxine (T4) into the more active triiodothyronine (T3) [38]. It exerts its effects on thyroid metabolism seleno-proteins, a group of biologically active protein molecules involved in diverse processes including DNA synthesis and thyroid metabolism [10]. Previous research has described the importance of adequate selenium levels for optimal thyroid function, highlighting how selenium deficiency can impair the conversion process, potentially leading to altered thyroid hormone levels and subsequent metabolic disturbances [39]. Moreover, clinical research suggests that selenium deficiency is linked with a higher risk of elevated anti-thyroid antibody levels, while selenium supplementation has been shown to reduce thyroid peroxidase antibody levels [40, 41]. Selenium status has also been correlated with Grave’s disease, autoimmune hypothyroidism and thyroid cancer [42]. In the context of adolescent Afghan refugees, who are likely exposed to a variety of environmental stressors and nutritional deficiencies, our findings of a positive association between T4 status and selenium levels suggest that selenium may play a crucial role in maintaining thyroid function amidst these challenges.

Although our study is the first of its kind assessing the association between micronutrients status and thyroid function in vulnerable refugee population, it has some limitations. First, due to limited sample size and cross-sectional design, the study findings cannot be generalized to other refugee and host populations. Secondly, we could not collect dietary data and other lifestyle factors which may also be associated with micronutrients status and thyroid profile in this population. Nonetheless, the study provides important baseline data information regarding the potential impact of micronutrients on thyroid profile in a vulnerable population.

## Conclusion

Overall, our study reports age and gender-based impacts of different micronutrients (both vitamins and minerals) on thyroid function in adolescent Afghan refugees. Of these, vitamin D and zinc are especially important as the serum level of these micronutrients is significantly correlated with thyroid hormone levels in this population. These findings highlight the importance of close monitoring and effective nutritional interventions to prevent thyroid related disorders in vulnerable population such as refugees.

## Electronic supplementary material

Below is the link to the electronic supplementary material.

Supplementary Material 1

## Data Availability

No datasets were generated or analysed during the current study.
