# Supplementary material for: Association of micronutrient status with thyroid function in adolescent Afghan refugees; a cross-sectional study
Source: Thyroid Res. 2025 Jun 3;18:23. doi: 10.1186/s13044-025-00239-6 (PMC12131714; doi:10.1186/s13044-025-00239-6)
Supplement: Supplementary file 1 — Supplementary Material 1 [file 13044_2025_239_MOESM1_ESM.docx]

Supplementary Table 1: Demographic and socio-economic characteristics of the study participants*

| Characteristics | Total | Male | Female | P-Value |
| --- | --- | --- | --- | --- |
| Age (Years) | 13.5±3.0/  13.0 (11.0, 16.0) | 13.3±2.9 | 13.7±3.1 | 0.27 |
| Age Cat (Years) |  |  |  |  |
| 10-14 | 121 (64.02%) | 66 (71.0) | 55 (57.3) | 0.05 |
| 15-19 | 68 (35.98%) | 27 (29.0) | 41 (42.7) |  |
| Educational attainment of the respondents |  |  |  |  |
| No formal Education | 36 (19.05%) | 9 (9.7) | 27 (28.1) | 0.002 |
| Primary Level | 113 (59.79%) | 59 (63.4) | 54 (56.3) |  |
| High school level | 37 (19.58%) | 22 (23.7) | 15 (15.6) |  |
| College & University | 3 (1.59%) | 3 (3.2) | - |  |
| Family size |  |  |  |  |
| 1-4 | 6 (3.17%) | 5 (5.4) | 1 (1.0) | 0.16 |
| 5-9 | 73 (38.62%) | 40 (43.0) | 33 (34.4) |  |
| 10-19 | 92 (48.68%) | 41 (44.1) | 51 (53.1) |  |
| 20 or more | 18 (9.52%) | 7 (7.5) | 11 (11.5) |  |
| Missing |  |  |  |  |
|  |  |  |  |  |
| Educational Attainment of Female (head/spouse) |  |  |  |  |
| No formal Education | 143 (75.66%) | 74 (79.57%) | 69 (71.88%) | 0.03 |
| Primary Level | 19 (10.05%) | 12 (12.90%) | 7 (7.29%) |  |
| High school level | 24 (12.70%) | 7 (7.53%) | 17 (17.71%) |  |
| College & University | 3 (1.59%) | 0 (0.00%) | 3 (3.13%) |  |
| Income quartiles |  |  |  |  |
| Q1 |  | 74 (79.6) | 69 (71.9) |  |
| Q2 |  | 12 (12.9) | 7 (7.3) |  |
| Q3 |  | 7 (7.5) | 17 (17.7) |  |
| Q4 |  | 74 (79.6) | 69 (71.9) |  |

**Age: mean±SD/median (Q1, Q3); Categorical variables: frequency (percentage); Different subscripts represent signifiant difference at 0.05 level; statistical test of significant difference: Kalmogorove Smirnove test for age and Chi-square and Fisher Exact tests for age categories and other variables respectively.*
